# Use of digital health interventions by outdoor workplaces in Australia: A focus on skin cancer prevention interventions

**DOI:** 10.1177/20552076261452162

**Published:** 2026-07-09

**Authors:** Christina Sandel, Janet McColl-Kennedy, Monika Janda

**Affiliations:** 1 Centre for Health Services Research, Faculty of Medicine, 1974 The University of Queensland, Woolloongabba, Queensland, Australia; 2 UQ Business School, Faculty of Business, Economics and Law, 1974 The University of Queensland, St Lucia, Queensland, Australia; 3 Institute of Health and Biomedical Research, School of Public Health and Social Work, Faculty of Health, Institute of Health and Biomedical Research, Queensland University of Technology, Brisbane, Queensland, Australia

**Keywords:** mHealth, occupational health, digital technology, skin cancer, digital health

## Abstract

**Background:**

Understanding digital health interventions (DHIs) from the user perspective is essential for developing best-practice, consumer-informed solutions.

**Objective:**

Australia has the highest incidence of skin cancer globally, yet awareness of how DHIs can mitigate its impact remains limited. This study explored the views of outdoor workplaces and workers in sectors such as construction, recreation, and water utilities across NSW and QLD regarding DHIs that promote sun protection and general wellbeing.

**Methods:**

A mixed-methods study recruited 15 outdoor workplaces and 107 workers to complete a REDCap survey using a snowball sampling approach. The survey for workplaces contained 10 closed and 11 open-ended questions. The survey for workers contained 16 closed and seven open-ended questions. Data were analysed using Excel, guided by the Capability, Opportunity, Motivation Behaviour (COM-B) framework to examine links between worker capability, opportunity, motivation, and awareness of DHIs.

**Results:**

Almost all participants (98/107; 91.6% outdoor workers) reported to have no or only limited exposure to digital health interventions that promote skin cancer awareness and sun exposure recommendations. Very few outdoor workplaces (6/15; 40%) and outdoor workers (21/107; 19.6%) had used other general health and wellbeing-focused digital health interventions. Outdoor workers raised they have very little understanding or awareness of what digital health interventions are, how they could be used and how individuals and workplaces can benefit from it. Outdoor workers raised their need for design elements that are specific to their work setting and environment.

**Conclusion:**

Findings suggest that outdoor workplaces need to consider implementing targeted digital health interventions to facilitate behaviour change to address a range of outdoor worker needs. Review of current digital health intervention design models, frameworks and strategies need to be undertaken for priority population groups such as outdoor workers. Current interventions do not appear to address user needs.

## Introduction

Frequent unprotected exposure to high ambient ultraviolet (UV) radiation from the sun causes skin cancer.^1^ The incidence of skin cancer in Australia is among the highest in the world, making skin cancer prevention a national public health priority.^2^ The cost burden of skin cancer to the Australian health system is approximately $1.7 billion per year,^2^ and further best-practice prevention-focused solutions are required to see a considerable impact on incidence rates.

High risk population groups such as outdoor workers are at higher risk of a skin cancer diagnosis due to frequent sun exposure during periods of high UV radiation.^3^ Outdoor workers are commonly defined as those who work a minimum of three hours outside, each day, and represent a diverse group of industries, including building and construction, recreational/sports, agriculture, mining, water utilities, physical education teachers and many others.

Sun protection behaviours of outdoor workers in their workplace setting are influenced by a number of factors including the provision of sun safety guidance in policies, protective equipment like long sleeved clothing and hats, and interventions that improve worker perceptions and behaviour.^4^ In particular, workplaces across Australia have a duty of care to reduce skin cancer incidence amongst their staff via the Australian Nuclear Protection and Nuclear Safety Agency’s Radiation Protection Standard for Occupational Exposure to Ultraviolet Radiation (2006).^5^ In addition to these standards, there are other state and national guidance documents that provide advice on sun protection requirements for outdoor workers.^6–9^ Peak non-government advocacy bodies like Cancer Councils also play an important role in delivering best practice sun protection guidance for outdoor workers in Australia.^10^ Workplaces can fulfill their requirements through stringent implementation of workplace health and safety policies and by communicating these policies and standards through best practice sun protection interventions that aim to minimise the impact of UV exposure on the outdoor worker’s health outcome.^11^ Surprisingly, in an international context, not all government bodies provide similar recommendations for outdoor workers. For example, the U.S. Preventative Services Task Force does not provide any specific recommendation about improving sun protection behaviours for outdoor workers but provides general guidance for the public.^12^

Digital health delivery methods are emerging as flexible solutions to deliver interventions through the use of digital technologies such as webpages, text messaging, mobile applications, emails and wearable devices.^13,14^ Digital health interventions have been successful in facilitating healthy behaviours, targeting issues such as supporting people to increasing physical activity, reduce/eliminate alcohol^15^ or smoking,^16,17^ and addressing chronic illnesses and mental health.^14^ Since COVID-19, there has been an increased interest in digital health interventions, particularly within the healthcare industry.^18^ Interestingly, the costs of implementing digital interventions are often lower than traditional program-based interventions,^11^ making them feasible solutions for a range of organisations.

Several digital health interventions have been developed to promote sun protection and prevent skin cancer. A systematic review conducted by Niu, Bhurosy and Heckman reported that digital health interventions have improved sun protection behaviours in people. However the review also found that while studies focused and delivered interventions to a range of populations, but not enough targeted interventions for outdoor workers who are especially vulnerable to receive excessive sun exposure.^19^

To date, there is limited research evidence available about whether and how digital health interventions have been considered for the delivery of workplace policy-informed education for the prevention of skin cancers in outdoor workers. Understanding the implementation and the impact of digital health interventions in outdoor workers could lead to the development of refined solutions to skin cancer prevention for this high-risk group, and ultimately, workplace policy success.

To add to the available evidence, this paper reports findings from a survey of outdoor workplaces and outdoor workers in Australia to understand whether they have implemented digital health interventions for promoting sun protection behaviours or other health-related issues, whether subjectively they experienced any benefits or disadvantages for these interventions, and what they considered as essential or critical components for future digital health-delivered skin cancer prevention interventions. The COM-B model is a behaviour change framework which proposes that focusing on capability (C), opportunity (O) and motivation (M) can influence and lead to behaviour (B) change in individuals.^20^ The COM-B model was employed in this study to understand the relationship of intervention impact and behaviour change.

## Methods

### Research questions

The study aimed to answer the following research questions:

Primary research question: How do outdoor workers and outdoor workplaces perceive digital health interventions for the promotion of sun protection behaviour in an outdoor work setting? What digital health intervention capabilities do they think are necessary?

Secondary research questions:

1) Have outdoor workers ever used a digital health intervention that promotes sun protection behaviour?
2) Have outdoor workplaces implemented a digital health intervention that promotes sun protection behaviour?
3) What are some of the benefits of using a digital health intervention that promotes sun protection behaviour?
4) What are some of the disadvantages of using a digital health intervention that promotes sun protection behaviour?
5) What other kinds of digital health interventions have outdoor workers and outdoor workplaces used for other health-related issues, and what were their benefits and disadvantages?

### Study design

This mixed-methods study combined qualitative and quantitative approaches to seek insights from outdoor workplaces and outdoor workers using a survey. Recruitment was via a snowball sampling approach which aimed to recruit a minimum of two and up to ten outdoor workplaces in Queensland (Qld) and New South Wales (NSW). Through this approach, over 80 outdoor workplaces across NSW and Qld were identified via an online search and contacted for voluntary participation in the study. Qld and NSW collectively represent geographic locations with high levels of skin cancer incidence in Australia.^21^

### Inclusion criteria

Participants eligible for the study were outdoor workplace representatives in NSW and Qld and their outdoor workers. Participants representing outdoor workplaces had to hold a senior or Work Health and Safety role so they could provide meaningful input to the survey. Outdoor workers were defined as those that worked at least a minimum of 3 hours a day performing tasks outside in outdoor industries like building, construction, water utilities, recreational/sports, landscaping and roadworks. All participants were required to read and respond in English.

### Exclusion criteria

Workplaces and workers who predominantly worked indoors were excluded from this study.

### Study population

A total of 22 organisations across NSW and Qld registered their interest to participate and 15 of them completed the survey. No interest was indicated by 28 organisations, and no contact was received from the remaining workplaces. In most of the workplaces, the nominated contact person had a Work Health and Safety background. An email with instructions and the survey links were provided to the contact person together with a separate document which included an invitation for outdoor workers to complete the survey for the contact person to distribute. Participating outdoor workplaces were encouraged to invite at least 10 workers to complete the outdoor worker survey while the nominated contact person was encouraged to complete the survey on behalf of their organisation.

The workplace survey was completed by a Work Health and Safety representative or a senior representative of the workplace with experience in that area or oversight of the business. Workplaces were then asked for the worker survey to be completed by outdoor workers from the respective participating outdoor workplace.

### The survey and survey development

The two online surveys were developed for distribution via RedCap platform and made available to interested outdoor workplaces^1^: one survey catered to the organisation, and^2^ the second survey catered to outdoor workers. Prior to being provided to interested organisations, surveys were pilot-tested with three The University of Queensland staff and three people who do not have English as a first language and modified based on their feedback prior to distribution.

Both surveys included closed questions (10 for workplaces and 16 for workers) with answer categories for yes/no responses, single and tick all that apply multiple choice responses, and Likert scale questions that assess frequency of using a digital health intervention and perception of sun protection behaviour at work. Open-ended questions (11 for workplaces and 7 for workers) allowed participants to express additional views regarding the use of digital health interventions promoting sun protection behaviours, and digital health interventions more generally that were not covered by the survey questions.

The quantitative component of the survey captured data on demographic characteristics, sun protection behaviour, and whether outdoor workplaces or outdoor workers used a digital health intervention for sun protection or general well-being, whether they thought there were advantages or disadvantages to the interventions, and what type of digital health intervention they would like to see from a pre-defined list of interventions. The qualitative component of the survey captured outdoor workplace and outdoor worker experience of using the digital health intervention and what capabilities they would like to see in a future intervention to address their needs.

Questions were designed in consultation with researchers at The University of Queensland staff. Open ended questions allowed participants to provide additional comments, and demographic questions were also asked (refer to supplementary questions for questions).

In accordance with the ethics application, participants were advised that they can complete the survey anonymously, therefore their and their organisation’s details remained confidential. Consent for participation in this anonymous surgery was obtained via the survey platform. The first page that participant saw provided the participant information and consent details. At the bottom of the page participants were advised that commencing the survey by clicking next indicates their consent to participate.

### Data analysis

This study gathered both quantitative and qualitative data from surveys. The statistical analysis was performed using Microsoft Excel, to compute the counts and percentages of demographic questions about the participating outdoor workplaces and outdoor workers, and their use and attitudes towards digital interventions. Responses to open ended questions were themed against the COM-B behaviour change model to support the qualitative analysis component of this study. Figure 1 (refer to supplementary material) presents the themes and their relationship to the COM-B behaviour change model.

Most questions were mandatory for completion except for the open-ended questions. Only one question relating to gender had an option for ‘prefer not to say’ and this was excluded from the analysis.

## Results

### Characteristics of outdoor workplaces

A total of 15 organisation representatives completed the survey. Of these, nine were from NSW and six from Qld. Organisations represented three industries: building and construction (8/15), recreation/sports (5/15), and water utility (2/15).

Participants were Executive Director (1/15), CEO (1/15), Manager (2/15), Coordinator (2/15) and WHS Representative (6/15). Of these personnel, 8/15 were responsible for their workplace policies, 5/15 were not responsible for their workplace policies, and 2/15 were unsure. Table 1 provides the characteristics of the outdoor workplaces by state.

**Table 1.** Outdoor workplace and outdoor worker characteristics by state.

Outdoor workplace characteristics	No. of participants n (%)		
	NSW n=9 (60)	QLD n=6 (40)	Total n=15 (100)
Organisation type			
Building and construction	4 (44)	4 (67)	8 (53)
Recreation/sports	4 (44)	1 (17)	5 (33)
Water utilities	1 (11)	1 (17)	2 (13)
Organisation representative			
CEO	1 (11)	0 (0)	1 (7)
Executive Director	0 (0)	1 (17)	1 (7)
Director	2 (22)	0 (0)	2 (13)
Manager	2 (22)	0 (0)	2 (13)
Engineer	0 (0)	1 (17)	1 (7)
Coordinator	2 (22)	0 (0)	2 (13)
WHS Representative (including Health and Safety Manager)	2 (22)	4 (67)	6 (40)
Responsible for workplace policies			
Yes	4 (44)	4 (67)	8 (53)
No	3 (33)	2 (33)	5 (33)
Unsure	2 (22)	0 (0)	2 (13)

Outdoor worker characteristics	No. of participants n (%)		
	NSW n=42 (39)	QLD n=65 (61)	Total n=107 (100)
Age			
Under 18 years	1 (2)	0 (0)	1 (1)
18-24 years	5 (12)	3 (5)	8 (7)
25-39 years	20 (48)	29 (45)	49 (46)
40-59 years	13 (31)	28 (43)	41 (38)
60 years+	3 (7)	5 (8)	8 (7)
Gender			
Male	36 (86)	50 (77)	86 (80)
Female	6 (14)	15 (23)	21 (20)
Organisation purpose			
Building and construction	12 (29)	14 (22)	26 (24)
Recreation/sports	29 (69)	10 (15)	39 (36)
Water utilities	0 (0)	40 (62)	40 (37)
Cleaning services	1 (2)	1 (2)	2 (2)
Occupation			
Builder/construction worker	8 (19)	9 (14)	17 (16)
Outdoor Educator	13 (31)	0 (0)	13 (12)
Lifeguard	0 (0)	2 (3)	2 (2)
Operator/Field worker/Officer/Scientist	6 (14)	27 (42)	33 (31)
Electrician	2 (5)	3 (5)	5 (5)
Manager	6 (14)	12 (18)	18 (17)
WHS Representative	0 (0)	2 (3)	2 (2)
Cleaner	0 (0)	1 (2)	1 (1)
Coordinator	4 (10)	3 (5)	7 (7)
Plumber	2 (5)	1 (2)	3 (3)
Engineer	1 (2)	4 (6)	5 (5)
Psychologist	0 (0)	1 (2)	1 (1)
Sun protection ranking			
Good	15 (36)	7 (42)	42 (39)
Fair	18 (43)	21 (32)	39 (36)
Needs improvement	9 (21)	17 (26)	26 (24)

### Characteristics of outdoor workers

Table 1 provides the characteristics of the outdoor workers by state. Overall, 107 outdoor workers completed the outdoor worker survey. The majority of workers were male (86/107, 80%); 65/107 (61%) respondents were from Qld and 42/107 (39%) from NSW. Workers represented four types of organisations: water utilities, recreation/sports, building and construction and cleaning. Most participating workers were 25 years or older. The occupation of workers varied, with representation of staff in junior and senior roles.

### Outdoor workplace results

#### Sun protection related digital health interventions

None of the 15 workplaces had implemented a sun protection digital health intervention and therefore no benefits or disadvantages of these types of interventions were reported.

#### General health and wellbeing related digital health interventions

Of the 15 workplaces, 6/15 (40%) had implemented a digital health intervention that promotes general health and wellbeing, with 4/15 (26.7%) of workplaces reported more than one intervention, while 9/15 (60%) participants had not implemented a digital intervention.

Of the 40% of workplaces (6/15) that had implemented a digital health intervention, 13% (2/15) used a health-related app for their workers, 13% (2/15) provided health-related text messages, 33% (5/15) referred to a form of health-related web program, 7% (1/15) provided awareness training through videos, and 7% (1/15) used weekly digital safety messages. Table 2 provides a breakdown by organisation type on these results.

**Table 2.** Types of general health and wellbeing digital health interventions implemented and preferred by workplaces.

Type of digital health intervention	No. workplaces that use intervention n (%)			No. workplaces that prefer a particular type of intervention n (%)		
	NSW n=9 (60)	QLD n=6 (40)	Total n=15 (100)	NSW n=9 (60)	QLD n=6 (40)	Total n=15 (100)
Text messaging						
Building and construction	0 (0)	1 (17)	1 (7)	1 (11)	1 (17)	3 (20)
Recreation/sports	1 (11)	0 (0)	1 (7)	1 (11)	0 (0)	1 (7)
Water utilities	0 (0)	0 (0)	0 (0)	0 (0)	0 (0)	0 (0)
Mobile application						
Building and construction	1 (11)	0 (0)	1 (7)	3 (33)	1 (17)	4 (27)
Recreation/sports	0 (0)	0 (0)	0 (0)	1 (11)	0 (0)	1 (7)
Water utilities	1 (11)	0 (0)	1 (7)	0 (0)	0 (0)	0 (0)
Web-based program						
Building and construction	1 (11)	2 (33)	3 (20)	0 (0)	0 (0)	0 (0)
Recreation/sports	1 (11)	0 (0)	1 (7)	0 (0)	0 (0)	0 (0)
Water utilities	1 (11)	0 (0)	1 (7)	0 (0)	0 (0)	0 (0)
Other – Audiovisual (video)						
Building and construction	1 (11)	1 (17)	2 (13)	n/a	n/a	n/a
Recreation/sports	0 (0)	0 (0)	0 (0)			
Water utilities	0 (0)	0 (0)	0 (0)			
Other – online platform Yammer						
Building and construction	0 (0)	0 (0)	1 (7)	n/a	n/a	n/a
Recreation/sports	1 (11)	0 (0)	1 (7)			
Water utilities	0 (0)	0 (0)	0 (0)			
Wearable device						
Building and construction	n/a	n/a	n/a	0 (0)	1 (17)	1 (7)
Recreation/sports				0 (0)	0 (0)	0 (0)
Water utilities				1 (11)	0 (0)	1 (7)
Not sure						
Building and construction	n/a	n/a	n/a	2 (22)	1 (17)	3 (20)
Recreation/sports				1 (11)	0 (0)	1 (7)
Water utilities				0 (0)	1 (17)	1 (7)

Examples of benefits of these general health and wellbeing interventions included the intervention supporting workplace health and culture, intervention had good functionality, intervention was easy to use, and intervention promoted positive behaviour change. Examples of disadvantages included issues with accessibility and practicality. Further details of these benefits and disadvantages are included in Supplementary Table 1.

#### Considerations regarding the development of a digital health intervention for skin cancer prevention

The most common digital intervention that workplaces were interested in implementing was a mobile application (46%). The second most interested digital health intervention was text messaging (13%). While some workplaces were not sure of what type of digital intervention they could implement (33%), all participating organisations showed interest in testing a future intervention of this kind within their workplace. A summary of these findings is provided in Table 2.

Workplaces raised a number of considerations for desired interventions, including ensuring it is practical, cost-effective and accessible, supports workplace health and culture, is supported by policy, promotes behaviour change, has a degree of safety, is innovative and has relevant content. A summary of workplace comments is provided in Supplementary Table 2.

General considerations regarding developing a digital health intervention included:

“For anything digital, quick and easy is often the key. Easy, robust” (Workplace representative #10)

“…wearable device is unlikely to be used due to being on roof...an App that audibly notifies the user of exposure risks would be helpful” (Workplace representative #22)

“Must relate to construction and include out of work hours, after hours and week-end exposure examples and information around annual skin checks.” (Workplace representative #2)

“Engaging and new technology” (Workplace representative #11)

“Useful information for the working environment that staff can connect with” (Workplace representative #10)

“One that is not costly” (Workplace representative #4)

Considerations regarding text messaging intervention included:

“I can see the value of such a system if workers are exposed to direct sunlight everyday or a few times a week.” (Workplace representative #7)

But, “Sending texts can be easily confused with spamming.” (Workplace representative #12)

“Never been available, but happy to pursue this further” (Workplace representative #8)

“Have not reached that level of maturity” (Workplace representative #2)

Our findings show that there was also a lack of understanding and or awareness of the potential usefulness of mobile phone related applications:

“Not thought of it. (Workplace representative #6)

And moreover, a negative view was expressed about the value of taking this approach. For example,

“The imposition of information through apps or texts can have a negative impact on the message being received. Not many people like being told what to do or how to do it” (Workplace representative #12)

### Outdoor worker results

#### Sun protection related digital health interventions

Of the 107 participants, 9/107 (8%) reported that they had used a sun protection digital health intervention in their workplace and one of these workers reported they used more than one intervention. Of these nine (9) workers, 2 (2%) used a mobile application, 1 (1%) a web-based program, 1 (1%) flash alerts, 1 (1%) used the ‘HammerTech’ platform to provide notifications and 5 (5%) used emails. Refer to Supplementary Table 3 for a breakdown of these results by state and organisation.

#### General health and wellbeing related digital health interventions

Sixteen of the 107 participants reported that they had used a form of digital health intervention that promotes general health and wellbeing. Most workers used a form of wearable device (20%), some used mobile application (12%), while none used text messaging-delivered intervention. Refer to Supplementary Table 4 for a breakdown of these results by state and organisation.

#### Considerations regarding the development of a digital health intervention for skin cancer prevention

While workers were not asked a specific question about the type of digital intervention they would like to see developed, some workers raised points in the additional comments box. A summary of these findings are provided in Supplementary Table 5. Considerations included those relating to intervention capability, intervention content, behaviour change and accessibility.

Interestingly, several workers expressed that they did not understand the potential benefit of digital intervention tools. For example, *“I have no idea what digital interventions are or how they work” (Outdoor worker #46*).

Some workers raised that they were not sure what this type of intervention would entail. Several highlighted the need to consider that outdoor workers do not always have access to network connectivity or mobile phones which can restrict their use of such interventions, as illustrated below.

“With mobility solutions now ubiquitous for works management, reminders or push notifications at the point of opening tasks on tablets or other devices would be a useful addition.” (Outdoor worker #72)

“If digital programs were to be used they would need to be able to operate both online and offline due to the nature of our workplace - often being away from data signals/wifi.” (Outdoor worker #5)

“…we often operate outside of areas of cellular reception and as such digital interventions (e.g. a reminder when UV hits a certain point) would potentially only be received days after the information was relevant. Analog methods such as a satellite phone call or in person communication are frankly more reliable in these circumstances” (Outdoor worker #107)

Further, an outdoor worker highlighted their limited knowledge of the benefits associated with wearing sun protective clothing, indicating the need for further education around benefits around sun protection behaviours.

“…but covering up means i not getting enough vitamin d naturally” (Outdoor worker #95)

## Discussion

### Sun protection and general-wellbeing related digital health interventions

This study sought to understand the current use, enablers and barriers for skin cancer prevention digital health interventions from the perspective of workplaces and outdoor workers. While some workplaces had implemented a digital intervention for general wellbeing, interestingly, none of them had implemented a sun protection digital health intervention. Similarly, only a small amount of outdoor workers had used a digital intervention relating to skin cancer prevention or general health and wellbeing, and they had very explicit needs for what a digital health intervention should look like. The breakdown of gender representation in this study is similar to how male and female outdoor workers are represented nationally, as there are more males than females in the outdoor workforce.^22^

### Considerations for development of digital health interventions for outdoor workplaces

Although literature suggests that multi-component health interventions are a successful solution to achieving healthy outcomes for workers,^23–25^ the demand for digital health interventions is increasing as digital technology is becoming an integral tool for people of all ages.^11^ Furthermore, implementation of digital health interventions in workplaces is considered low cost and easily scalable.^11^ These key drivers continue to increase its popularity amongst workplaces; however, not all workplaces are equipped with knowledge and understanding of digital interventions. This may also have a direct correlation to the type of work being done, or the maturity of the workplace.

A systematic review concluded that digital interventions are promising to deliver sun protection interventions and improve behavioural outcomes for people.^19^ However, this study found a number of barriers for such interventions in the context of outdoor workplaces that still need to be overcome. Our study demonstrated that while outdoor workplaces were interested in implementing digital health interventions, particularly via a mobile phone, they did not understand how sun safe policies could be distributed, and what their potential impact may be if implemented. There appears to be a significant gap in organisations awareness regarding the capabilities and application of digital health solutions. In particular, outdoor workplaces have very limited awareness of how they could adopt solutions that exist for skin cancer prevention.^26^ As research continues to validate the behaviour change impact of digital health interventions,^11^ further advocacy is required to promote digital interventions to all types of workplaces. There is a key role that peak bodies and governments can play to ensure that guidelines, policies or strategies identify and recommend best practice interventions to workplaces to assist with the prevention of skin cancer. This will need to be accompanied by a model that suggests how cost-effective digital health interventions can be adopted across small-scale and large-scale businesses. Interventions need to be focused on maintaining workplace safety,^27^ while also continuing to optimise organisational health-related objectives.

### Considerations for the development of digital health interventions for outdoor workers from a behavioural change perspective using the COM-B model

Critical to the implementation success of a digital health intervention is its ability to meet the needs of its users.^28^ The COM-B framework can help digital health intervention designers understand what elements of a digital intervention can influence Capability, Opportunity and Motivation to influence an outdoor workers behaviour.^29^

In alignment with the *Capability* aspect of the COM-B model, there is a significant correlation between physical and psychological capabilities of outdoor workers and their level of awareness regarding digital health interventions that support the prevention of skin cancer.^26^ Our study findings suggest that the lack of outdoor workplace knowledge of digital health interventions will impact knowledge in their workers and their ability to improve or adopt sun protection practices.^30^ This barrier will need to be addressed through advocacy and maturing organisations but also through building public awareness of available tools through outreach and communication best led by relevant government agencies such as the Australian Digital Health Agency and other state level departments. Psychological capability can also be impacted by outdoor workers receiving too much information, leading them to react negatively toward the messaging and disrupting potential behaviour change.^31^

In alignment with the *Opportunity* aspect of COM-B model, due to the nature of their work, outdoor workers desire an intervention that can be accessible regardless of location and without an internet connection. This remains a critical opportunity barrier which reduces user ability to adopt sun protection behaviour. Sugg et al. (2020) concluded that outdoor workers using a wearable device were more likely to change their behaviour when notifications were received regarding heat related conditions.^32^

Designers need to consider the various working environments of outdoor workers and ensuring that geographic location does not inhibit outdoor worker interaction with the intervention.

Literature confirms that the general population prefers to use web-based interventions on mobile phones or computers to access skin cancer related interventions due to flexibility and design features. However, this is determined on an individual’s browsing behaviour.^19^ In alignment with the *Motivation* aspect of COM-B model, outdoor workers are most interested to see intervention content, intervention capability and intervention accessibility as key components of a future intervention. Content should be tailored and consistent with workplace policy and government advice.^33,34^ Importantly, intervention should be capable in its design and have versatile functionality, in particular having interactive capabilities. Developers can view these intervention components as user recognition of what will motivate them to use a digital health intervention.

### Future research

This survey has provided important insights for future development and refinement of digital health interventions for outdoor workers. Critically, it has indicated the current design gap that has led to lack of digital health solutions adoption for improving outdoor workers’ sun safety. To address this gap, further research is required into whether policies, strategies and recommendations specifically for designing and developing digital health interventions for outdoor workers exist, and what design elements they suggest specifically for this high-risk population group, and whether they align with the workplaces and worker needs. Further advocacy work is also required for development and implementation of co-designed digital health interventions for outdoor workers in outdoor workplaces by policy makers, outdoor workplaces, unions and peak bodies. The results of this survey will be used in the future to develop a co-designed framework for digital health interventions that can successfully communicate skin cancer prevention messages to outdoor workers.

## Limitations

This was an exploratory study which adopted a snowball sampling approach to recruit participants from New South Wales and Queensland. There are various outdoor worker industries across Australia who have not participated in this study; therefore, the participants may not be reflective of a wider national or international cohort of outdoor workers. While qualitative and quantitative data was collected under a mixed-methods study, the focus of the study was not to make many inferences across the data as this will be explored in a larger research.

## Conclusion

The findings of this survey indicate that while digital health interventions have been implemented for various population groups, there are limited interventions that have been developed and implemented for outdoor workers to support skin cancer prevention efforts and promote general health and wellbeing. This high-risk population group has specific needs that have restricted their ability to either access or understand how to use or receive clear messaging from a digital health intervention, particularly interventions specific to skin cancer prevention. Intervention capability and content are two key areas requiring personalisation to specific outdoor workplaces. However, the development of such interventions need to be affordable to outdoor workplaces of all sizes or very limited outdoor workers will receive the benefit of using digital health interventions in efforts to prevent skin cancer. In conclusion, our study provides a solid grounding on which future studies can build. We encourage future research into this important and timely topic.

## Supplemental material

Supplemental material - Use of digital health interventions by outdoor workplaces in Australia: A focus on skin cancer prevention interventions Supplemental material for Use of digital health interventions by outdoor workplaces in Australia: A focus on skin cancer prevention interventions by Christina Verma, Janet McColl-Kennedy, and Monika Janda in DIGITAL HEALTH.

Supplemental material - Use of digital health interventions by outdoor workplaces in Australia: A focus on skin cancer prevention interventions Supplemental material for Use of digital health interventions by outdoor workplaces in Australia: A focus on skin cancer prevention interventions by Christina Verma, Janet McColl-Kennedy, and Monika Janda in DIGITAL HEALTH.

## Data Availability

Data can be requested from the corresponding author.*
