# Supplementary material for: Use of digital health interventions by outdoor workplaces in Australia: A focus on skin cancer prevention interventions
Source: Digit Health. 2026 Jul 9;12:20552076261452162. doi: 10.1177/20552076261452162 (PMC13351230; doi:10.1177/20552076261452162)
Supplement: Supplemental material - Use of digital health interventions by outdoor workplaces in Australia: A focus on skin cancer prevention interventions [file sj-pdf-1-dhj-10.1177_20552076261452162.pdf]

Use of digital health interventions by outdoor workplaces in Australia: A focus on skin cancer prevention interventions

Supplementary material

Figure 1: COM-B framework application

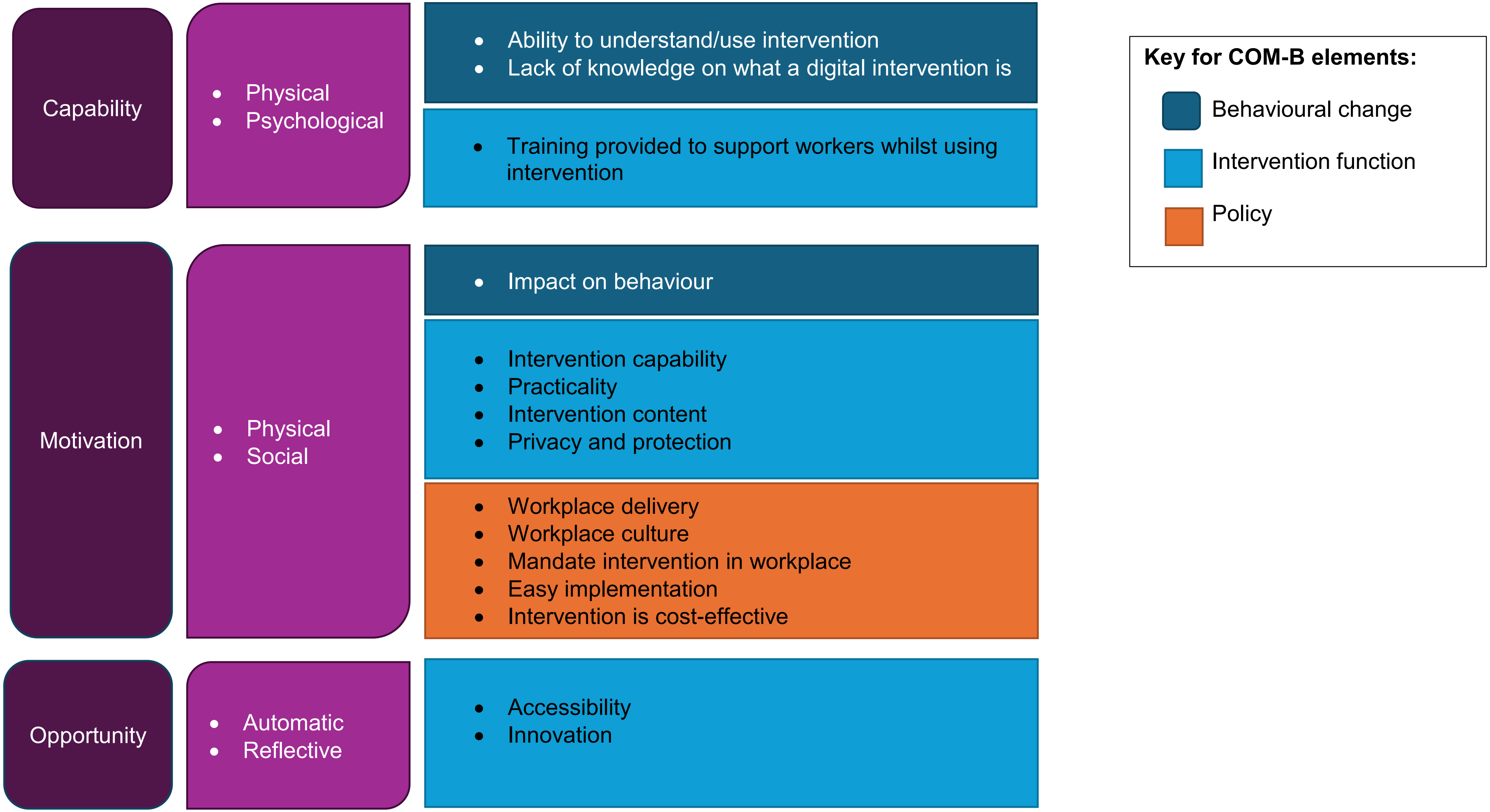

Table 1: Benefits and disadvantages of general health and wellbeing digital interventions implemented by workplaces.

Type of digital health intervention	Organisation type	Examples of benefits (linked to COM-B framework)	Examples of disadvantages (linked to COM-B framework)
**Mobile application**	Building & construction	N/A	One workplace stated that there were no benefits to using this intervention
	Water utilities	**Workplace culture** - e.g. "*Supported team building and improving staff wellbeing*"	**Accessibility** – e.g. *"A minority of staff are unable to access their mobile device while physically working (devices are kept in vehicles)"* **Practicality** – e.g. *"Other workers prefer to not use technology due to being saturated by it while some of the older staff who prefer hard copy information"*
**Text messaging**	Building & construction	**Intervention capability** – e.g. *"Consistent messaging language and format"*	N/A
**Web-based program**	Building & construction	**Intervention capability** – e.g. *"Consistent messaging language and format"*	N/A
	Water utilities	**Practicality** – e.g. *"Great way to get information out to more than just the core staff team"*	**Practicality** – e.g. "*Some have found "Workplace" difficult to use on their mobile devices"*
	Recreation/sports	**Impact of behaviour** – e.g. "*Increased encouragement of health lifestyle and attitude"*	N/A
**Other – audiovisual (video)**	Building & construction	**Intervention capability** – e.g. "*Consistent messaging language and format"*	N/A
**Other – online platform**	Water utilities	**Intervention capability** – e.g. *"Constant reminders"*	N/A

(Yammer)			

Table 2: Considerations raised by outdoor workplaces for the development of a digital health intervention promoting skin cancer awareness.

Outdoor workplace type	State	Considerations to developing an intervention	Considerations regarding text messaging intervention	Considerations regarding mobile phone related applications
**Building and construction**	NSW	• **Lack of knowledge** – e.g. "Not sure" (Workplace representative #1)<br>• **Intervention content –** e.g. "Exposure time warning" (Workplace representative #22)<br>• **Accessibility and practicality** – e.g. "…wearable device is unlikely to be used due to being on roof...an App that audibly notifies the user of exposure risks would be helpful" (Workplace representative #22)<br>• **Workplace culture** – e.g. "Less force, more encouragement so workers don't feel imposed"	• **Lack of knowledge and intervention content** – e.g. "I don't know how big of a issue it could of been and the type of information that would of been required to be promoted" (Workplace representative #1)<br>• **Workplace culture and mandate intervention in workplace** – e.g. "We don't often work in direct sunlight but if we do we often discuss staying hydrating and safe however I can see the value of such a system if workers are exposed to direct	• **Lack of knowledge** – e.g. "Not sure. Never came across it" (Workplace representative #1)<br>• **Impact on behaviour and ability to understand/use intervention** – e.g. "The imposition of information through apps or texts can have a negative impact on the message being received. Not many people like being told what to do or how to do it" (Workplace representative #12)

		(Workplace representative #12)	sunlight everyday or a few times a week." (Workplace representative #7)<br>• **Privacy and protection** – e.g. "Sending texts can be easily confused with spamming." (Workplace representative #12)<br>• **Impact on behaviour** – e.g. "…Simple apps like teams when broadcasting has the potential to annoy workers" (Workplace representative #12)	
	QLD	• **Intervention content** – e.g. "Must relate to construction and include out of work hours, after hours and week-end exposure examples and information around annual skin checks." (Workplace representative #2)<br>• **Intervention content and capability** – e.g. "Notification of temperatures and UV rating for the day." (Workplace	• **Workplace culture** – e.g. "have not reached that level of maturity" (Workplace representative #2)<br>• **Privacy and protection** – e.g. "Too much spam already" (Workplace representative #11)<br>• **Workplace culture** – e.g. "never been available, but happy to pursue this further" (Workplace representative	• **Workplace culture** – e.g. "…technology has not been available" (Workplace representative #8)

		representative #3)<br>• **Innovation** – e.g. "Engaging and new technology" (Workplace representative #11)	#8)	
**Water Utility**	NSW	• **Intervention capability** – e.g. "1.easy to use - quick to put on/turn on 2large print (in case one forgets glasses) 3.light weight 4.water proof 5.does 2 things not just one thing - for eg highlights sun exposure factors+ temperature +dehydration levels of wearer)" (Workplace representative #17)<br>• **Workplace culture** – e.g. "Digital interventions can quickly become obsolete if not designed well they can also inherit a bad reputation if they provide short cuts to optimum WHS processes undermining other objectives" (Workplace representative #17)<br>• **Intervention capability** – e.g. "…Needs to be simple to use and not interfere with work	• N/A	• N/A

		being completed" (Workplace representative #19)		
	QLD	• **Intervention content** – e.g. "Exposure hours, time of day" (Workplace representative #13)	• N/A	• **Lack of knowledge** – e.g. "Unaware of what is available" (Workplace representative #13)
**Recreation and sports**	NSW	• **Intervention capability** – e.g. "For anything digital, quick and easy is often the key. Easy, robust" (Workplace representative #10)<br>• **Intervention content** – e.g. "Useful information for the working environment that staff can connect with" (Workplace representative #10)	• N/A	• **Workplace culture and ability to understand/use intervention** – e.g. "It had not been something that we had considered prior to this survey. Now that we are a part of this, it is something that we would consider. Also, digital applications are not something that are really used in the outdoor education environment. We try to use the time we have with our clients to step away from digital platforms and enjoy being out in nature." (Workplace representative #10)<br>• **Lack of knowledge** – e.g. "Not

				thought of it. (Workplace representative #6)<br>• I'm not sure what these might look like, other than messages reminding people to follow sun protection guidelines" (Workplace representative #9)
	QLD	• **Intervention is cost effectiveness** – e.g. "One that is not costly" (Workplace representative #4)<br>• **Intervention capability and workplace culture/policy-** e.g. "easy to implement" (Workplace representative #4)	• N/A	• **Workplace culture/delivery** – e.g. "Not really a sun safety aspect, but one of our worksite used a 'pre-work' risk assessment app (for a mine site)." (Workplace representative #4)

Table 3: Types of sun protection digital health interventions used by outdoor workers.

Type of digital health intervention	No. workers that have used intervention n (%)		
	NSW n=42 (39)	QLD n=65 (61)	Total n=107 (100)
Mobile application			
• Building and construction	0 (0)	0 (0)	0 (0)
• Recreation/sports	2 (5)	0 (0)	2 (2)
• Water utilities	0 (0)	0 (0)	0 (0)

• Cleaning	0 (0)	0 (0)	0 (0)
Web-based program			
• Building and construction	0 (0)	0 (0)	0 (0)
• Recreation/sports	0 (0)	0 (0)	0 (0)
• Water utilities	0 (0)	1 (2)	1 (1)
• Cleaning	0 (0)	0 (0)	0 (0)
Other – Flash alerts			
• Building and construction	0 (0)	0 (0)	0 (0)
• Recreation/sports	0 (0)	0 (0)	0 (0)
• Water utilities	0 (0)	1 (2)	1 (1)
• Cleaning	0 (0)	0 (0)	0 (0)
Other – Hammertech notification			
• Building and construction	1 (2)	0 (0)	1 (2)
• Recreation/sports	0 (0)	0 (0)	0 (0)
• Water utilities	0 (0)	0 (0)	0 (0)
• Cleaning	0 (0)	0 (0)	0 (0)
Other – Email			
• Building and construction	0 (0)	0 (0)	0 (0)
• Recreation/sports	1 (2)	0 (0)	1 (1)
• Water utilities	0 (0)	3 (5)	3 (3)
• Cleaning	0 (0)	1 (2)	1 (1)

Table 4: Types of general health and wellbeing digital health interventions used by outdoor workers.

Type of digital health intervention	No. workers that have used intervention n (%)

	NSW n=42 (39)	QLD n=65 (61)	Total n=107 (100)
Wearable device			
• Building and construction	2 (5)	4 (6)	6 (6)
• Recreation/sports	2 (5)	0 (0)	2 (2)
• Water utilities	0 (0)	13 (20)	13 (12)
• Cleaning	0 (0)	0 (0)	0 (0)
Text messaging			
• Building and construction	0 (0)	0 (0)	0 (0)
• Recreation/sports	0 (0)	0 (0)	0 (0)
• Water utilities	0 (0)	0 (0)	0 (0)
• Cleaning	0 (0)	0 (0)	0 (0)
Mobile application			
• Building and construction	1 (2)	3 (2)	4 (4)
• Recreation/sports	1 (2)	1 (2)	2 (2)
• Water utilities	0 (0)	9 (14)	9 (8)
• Cleaning	0 (0)	0 (0)	0 (0)
Web-based program			
• Building and construction	0 (0)	1 (2)	1 (1)
• Recreation/sports	0 (0)	2 (3)	2 (3)
• Water utilities	0 (0)	5 (8)	5 (5)
• Cleaning	0 (0)	0 (0)	0 (0)
Other – Health assessment questionnaire			
• Building and construction	0 (0)	0 (0)	0 (0)
• Recreation/sports	0 (0)	1 (2)	1 (1)
• Water utilities	0 (0)	0 (0)	0 (0)

• Cleaning	0 (0)	0 (0)	0 (0)

Table 5: Considerations raised by outdoor workers for the development of a digital health intervention promoting skin cancer awareness

Organisation type	State	Considerations to developing a digital intervention
Building and construction	NSW	• **Lack of knowledge** – e.g. "…Unfamiliar with the style and application of such interventions." (Outdoor worker #97)
	QLD	• **Intervention capability and content** - e.g. "A great incentive that in my opinion will be more of a "reminder by repetition". Particularly relevant in the construction industry" (Outdoor worker #67)<br>• **Impact on behaviour** – e.g. "We normally take it upon ourselves to check the UV index on google every morning via smartphone/the work tablet." (Outdoor worker #31)
Water Utility	NSW	• **Lack of knowledge** – e.g. "…I'm not aware of it." (Outdoor worker #6)
	QLD	• **Intervention capability** – e.g. "With mobility solutions now ubiquitous for works management, reminders or push notifications at the point of opening tasks on tablets or other devices would be a useful addition." (Outdoor worker #72)<br>• **Lack of knowledge** – e.g. "I have no idea what digital interventions are or how they work" (Outdoor worker #46)<br>• **Intervention content** – e.g. "They may capture some audiences if they were to link to some reputable science on such matters, otherwise it's kind of stating the obvious" (Outdoor worker #1); "Other than the longstanding slip slop slap advise, there does not seem to be any further need" (Outdoor worker #22)
Recreation and sports	NSW	• **Lack of knowledge** – e.g. "…but covering up means i not getting enough vitamin d naturally" (Outdoor worker #95)<br>• **Intervention capability and accessibility** – e.g. "If digital programs were to be used they would need to be able to operate both online and offline due to the nature of our workplace - often being away from data signals/wifi." (Outdoor worker #5)<br>• **Intervention capability** – e.g. "Its too easy to try and use a digital intervention to find a result that will be either too vague or wrong, where a human can ask more questions based on your symptoms and can run tests to make a informed result" (Outdoor worker #105)<br>• **Accessibility** – e.g. "…we often operate outside of areas of cellular reception and as such digital interventions (E.g. a reminder when UV hits a certain point) would potentially only be received days after the information was relevant. Analog methods such

		as a satellite phone call or in person communication are frankly more reliable in these circumstances” (Outdoor worker #107)
	QLD	• N/A
