# Supplementary material for: Use of digital health interventions by outdoor workplaces in Australia: A focus on skin cancer prevention interventions
Source: Digit Health. 2026 Jul 9;12:20552076261452162. doi: 10.1177/20552076261452162 (PMC13351230; doi:10.1177/20552076261452162)
Supplement: Supplemental material - Use of digital health interventions by outdoor workplaces in Australia: A focus on skin cancer prevention interventions [file sj-pdf-2-dhj-10.1177_20552076261452162.pdf]

SURVEY ONE: OUTDOOR WORKERS

Survey title: Are digital health interventions currently being used in an outdoor worker setting?

Survey consent: By clicking on the next button, you acknowledge that you have read the participant information sheet, and you consent to participate in the survey.

Survey questions: Outdoor workers

Pre-survey questions

1. What gender do you identify as?
    - Male
    - Female
    - Non-binary
    - Prefer not to say
2. What is your age group?
    - Under 18
    - 18-24
    - 25-39
    - 40-59
    - 60 +
3. What state do you work in?
    - QLD
    - NSW
4. What is the main purpose of the organisation you work in?
    - Mining
    - Building and Construction
    - Agriculture
    - Telecommunications
    - Roadwork
    - Postal
    - Outdoor events
    - Recreation/Sports
    - Other
        - _________
5. What is your occupation?
    - Miner
    - Builder/Construction worker
    - Agricultural/farming worker
    - Telecommunication worker
    - Electrician
    - Plumbing

- Road worker
- Teacher
- Athlete
- Lifeguard
- Other
  - _________

Survey questions

1. How would you rate your sun protection behaviour at work?
   - Needs improvement
   - Fair
   - Good

The following questions refer to the use of a digital health intervention. Please note that a digital health intervention uses digital devices such as smartphones, tablets, portable devices or computers to deliver information/support with the aim to support outdoor workers to achieve good health and wellbeing. This may be through mobile applications, websites or text messaging.

2. Has your workplace ever supplied a digital health intervention that encourages sun protection behaviour?
   - No
   - Yes
     - What was the digital sun protection intervention?
       - _____________
     - Did you benefit from using this digital health intervention?
       - No
       - Yes
         - What were the benefits of using this digital health intervention?
           - ________
       - Unsure
     - Were there any disadvantages in using the digital health intervention?
       - No
       - Yes
         - What were the disadvantages of using this digital health intervention?
           - ________
       - Unsure
     - How often did you use this digital sun protection intervention?
       - Daily
       - Over several weeks
       - Over several months
       - Once-off
       - Other
     - Would you use this digital sun protection intervention again?
       - Yes
       - No

3. Please provide any additional comments you want to make about digital sun protection in the workplace.
    - ____

4. Do you use any other digital health intervention to improve your health?
    - No
    - Yes
        - What kind of digital intervention do/have you use/used? (tick all that apply)
            - Wearable devices such as a FitBit, smartwatch or step counter
            - Health related app
            - Receiving health related text messages
            - Health related web program (e.g. online gym sessions, online dieting programs etc)
            - Other
        - Were there any benefits in using this/these digital health interventions?
            - No
            - Yes
                - What were the benefits in using this/these digital health interventions?
                    - ________
            - Unsure
        - Where there any disadvantages in using this digital health intervention?
            - No
            - Yes
                - What were the disadvantages in using this digital health intervention?
                    - ________
            - Unsure
    - How often do/did you use this digital health intervention?
        - Daily
        - Over several weeks
        - Over several months
        - Once-off
        - Other

5. Please provide any additional comments you want to make about health related digital interventions in the workplace.
    - ____

Thank you for participating in this survey.

SURVEY TWO: OUTDOOR WORKPLACES

Survey title: Are digital health interventions currently being used in an outdoor worker setting?

Survey consent: By clicking on the next button, you acknowledge that you have read the participant information sheet, and you consent to participate in the survey.

Interview questions: Outdoor Workplace

Pre-survey questions

1. Which state is your organisation located?
   - QLD
   - NSW
2. What is the main purpose of your organisation you work in?
   - Mining
   - Building and Construction
   - Agriculture
   - Telecommunications
   - Roadwork
   - Postal
   - Outdoor events
   - Recreation/Sports
   - Other
     - __________
3. What is your position within the organisation?
   - _________
     - Are you responsible for sun safety policies and procedures in the organisation?
       - _________

Survey questions

The following questions refer to the use of a digital health intervention. Please note that a digital health intervention uses digital devices such as smartphones, tablets, portable devices or computers to deliver information/support with the aim to support outdoor workers to achieve good health and wellbeing. This may be through mobile applications, websites or text messaging.

1. Has your organisation ever implemented a digital health intervention that encourages sun protection behaviour?
   - No
   - Yes
     - What kind of digital sun protection health intervention was this?

- ________
- Were there any benefits of using this digital health intervention?
    - No
    - Yes
        - What were the benefits?
            - ________

2. Please provide any comments you want to make about sun protection in the workplace.
    - ____

3. Has your organisation ever implemented a digital health intervention to improve health and wellbeing of your staff?
    - No
    - Yes
        - What kind of digital intervention did you implement?
            - _______
        - Were there any benefits of using this digital health intervention?
            - No
            - Yes
                - What were the benefits of using this digital health intervention?
                    - ________
        - Were there any disadvantages to using this digital health intervention?
            - No
            - Yes
                - What were the disadvantages to using this digital health intervention?
                    - ________
4. Would you be interested in testing a digital health intervention that encourages sun protection behaviour with your workers?
    - No
    - Yes
        - What kind of intervention would you consider testing?
            - _______
        - What elements would you consider important for a digital intervention to be useable in your workplace?
            - _______
        - Have you ever used or considered using an intervention that involves sending text messages to your workers to promote their sun safety?
            - Yes
            - No
                - What is you reason for not considering this?
        - Have you ever used or considered using an intervention that involves your workers using a mobile application to promote their sun safety?
            - Yes
            - No

- What is you reason for not considering this?

5. Please provide any additional comments you want to make about health-related digital interventions in the workplace.
   ____

Thank you for participating in this survey.
